# Increased Tau Phosphorylation and Tau Truncation, and Decreased Synaptophysin Levels in Mutant BRI_2_/Tau Transgenic Mice

**DOI:** 10.1371/journal.pone.0056426

**Published:** 2013-02-13

**Authors:** Holly J. Garringer, Jill Murrell, Neeraja Sammeta, Anita Gnezda, Bernardino Ghetti, Ruben Vidal

**Affiliations:** Department of Pathology and Laboratory Medicine and Indiana Alzheimer Disease Center, Indiana University School of Medicine, Indianapolis, Indiana, United States of America; Dulbecco Telethon Institute and Mario Negri Institute for Pharmacological Research, Italy

## Abstract

Familial Danish dementia (FDD) is an autosomal dominant neurodegenerative disease caused by a 10-nucleotide duplication-insertion in the *BRI_2_* gene. FDD is clinically characterized by loss of vision, hearing impairment, cerebellar ataxia and dementia. The main neuropathologic findings in FDD are the deposition of Danish amyloid (ADan) and the presence of neurofibrillary tangles (NFTs). Here we investigated tau accumulation and truncation in double transgenic (Tg-FDD-Tau) mice generated by crossing transgenic mice expressing human Danish mutant *BRI_2_* (Tg-FDD) with mice expressing human 4-repeat mutant *Tau-P301S* (Tg-Tau). Compared to Tg-Tau mice, we observed a significant enhancement of tau deposition in Tg-FDD-Tau mice. In addition, a significant increase in tau cleaved at aspartic acid (Asp) 421 was observed in Tg-FDD-Tau mice. Tg-FDD-Tau mice also showed a significant decrease in synaptophysin levels, occurring before widespread deposition of fibrillar ADan and tau can be observed. Thus, the presence of soluble ADan/mutant BRI_2_ can lead to significant changes in tau metabolism and synaptic dysfunction. Our data provide new *in vivo* insights into the pathogenesis of FDD and the pathogenic pathway(s) by which amyloidogenic peptides, regardless of their primary amino acid sequence, can cause neurodegeneration.

## Introduction

Two early-onset autosomal dominant diseases known as familial British dementia (FBD) and familial Danish dementia (FDD) are caused by mutations in the *BRI_2_* gene [1]–[4]. FBD was first reported by Worster-Drought and McMenemey in 1933, who described a British kindred with presenile dementia and spastic paralysis [1]. FDD was first described by Strömgren and collaborators in 1970, who described a Danish kindred with predominant clinical features of vision impairment, hearing loss, and progressive dementia [2]. The *BRI_2_* gene (also known as *ITM2b*) encodes a protein of 266 amino acids that belongs to a family of integral type II trans-membrane domain proteins [3]–[6]. Cleavage of the BRI_2_ protein within its ectodomain (residue Arg243 and Glu244, KGIQKR^↓^EAS) by pro-protein convertases (PCs) [7]–[9] releases a 23 amino acid pro-peptide from the wild-type precursor protein and 34 amino acid long amyloid peptides in patients with FBD (ABri) and FDD (ADan) [3], [4]. A large part of the remaining ectodomain, the BRICHOS domain, is shed by ADAM10 and released into the extracellular space. The remaining membrane associated N-terminal fragment (NTF) undergoes intramembrane proteolysis mediated by SPPL2a or SPPL2b. This cleavage generates an intracellular domain, which is liberated into the cytosol and a secreted C domain [10]–[12].

The clinical phenotype of FBD and FDD consists of progressive dementia and ataxia. The main neuropathologic findings are the presence of severe amyloid angiopathy and neurofibrillary tangles (NFTs). Patients with FBD have hippocampal extracellular perivascular amyloid plaques and intraneuronal formation of NFTs within the limbic regions. Amyloid plaques are primarily localized to the hippocampus and cerebellum. NFTs are numerous in the hippocampus. Extensive cerebral amyloid angiopathy (CAA) is observed in the leptomeningeal vessels and in gray and white matter vessels within the cerebrum, cerebellum, brainstem, and spinal cord; however, cerebral hemorrhage rarely occurs in FBD patients [3], [13]. Neuropathologic findings of FDD closely resemble those of FBD; however, parenchymal deposits found in the hippocampus of patients with FDD are Congo red and Thioflavine S (ThS) negative. In addition, amyloid β (Aβ) can be found co-deposited with the Danish amyloid [4], [14]. Interestingly, tau deposits in FBD, FDD, Alzheimer disease (AD), and some forms of prion diseases are antigenically, ultrastructurally and biochemically similar [13]–[17]. NFTs in patients with FBD and FDD are composed of paired helical filaments (PHFs) as in patients with AD. Biochemical analyses of insoluble tau from FBD, FDD, and AD brains show a similar Western blot banding profile. The similarity between pathological lesions supports a unifying pathologic mechanism by which different amyloidogenic peptides could trigger a complex pathological cascade from soluble to filamentous insoluble tau protein [18].

Hyperphosphorylation and caspase-mediated truncation of tau at aspartic acid (Asp) residue 421 (Asp 421 or D421, longest human tau isoform) appears to play an important role in the assembly of tau into filaments [19], [20]. In fact, several *in vitro* and *in vivo* studies suggest that caspase activation might be an early event in NFT formation [19]–[21]. Using *in vivo* multiphoton imaging, it was observed that fibrillar tau deposits are the consequence of a cellular degenerative process marked by caspase activation [22]. After the formation of a new tangle within the neuron, the cell remained alive and caspase activity seems to be suppressed. Importantly, NFT formation and cleavage of tau at amino acid Asp 421 can be triggered by Aβ peptides, linking amyloid and tau [19].

Herein, we generated double transgenic (Tg-FDD-Tau) mice by crossing transgenic mice expressing human Danish mutant BRI_2_ (Tg-FDD) with mice expressing human 4-repeat mutant Tau-P301S (Tg-Tau) to study *in vivo* the relationship between BRI_2_, ADan and tau. Our studies provide novel *in vivo* insights into the pathogenesis of FDD and a mechanistic link between ADan, tau, and synaptic pathology.

## Materials and Methods

### Transgenic Mice

Transgenic (Tg) mice homozygous for human *BRI_2_* containing the 10 nucleotide duplication (787_796dupTTTAATTTGT) found in patients with FDD (Tg-FDD) [4], [23], mice expressing the smallest 4-repeat human isoform of the microtubule-associated protein tau (MAPT) (0N4R) with the frontotemporal dementia with parkinsonism linked to chromosome 17 (FTDP-17) P301S mutation (Tg-Tau) [24], [25], double homozygous Tg-FDD×Tg-Tau (Tg-FDD-Tau) [18] mice, and wild-type (WT) C57BL/6J mice were used. The P301S mutation was introduced into the smallest exon 10 containing *MAPT* cDNA by site-directed mutagenesis and ligated into XhoI digested pMoPrP.Xho vector [23]. The positive clones were analyzed for proper orientation and sequence. The resultant DNA was digested with NotI and given to the Indiana University Transgenic Core Facility for injection. The construct was free of all vector sequences prior to injection. Standard technique was used to generate the transgenic mice using C3HeB/FeJ mice. Transgenic pups were identified by amplifying tail DNA with human specific *MAPT* primers, 5′-CTCCAAAATCAGGGGATCGC-3′ and 5′-CCTTGCTCAGGTCAACTGGT-3′. Eight founders were mated with C57BL/6 mice. Copy number and integration of the transgene was determined by Southern blotting of tail DNA from founder offspring and expression of the transgene was determined by Northern analyses. The offspring were backcrossed to C57BL/6 mice to establish lines. Tg-FDD-Tau mice were generated by mating Tg-FDD and Tg-Tau mice. Expression of the transgenes (*BRI_2_* and *MAPT*) is under the control of the murine *Prnp* promoter [18], [23], [24]. Tg-FDD-Tau and single Tg-FDD and Tg-Tau mice have the same genetic background: C57BL/6. The presence of the transgenes was detected by PCR amplification as described [23], [24]. Body weight, coat appearance, posture, and tail suspension response were assessed in all mice as described [23], [26]. Coats were deemed to be either normal (smooth and clean) or rough (matted and dirty) by observing the fur at the back of the neck. A hunched or arched posture that was apparent while the mouse was both sitting and walking was noted. The tail suspension response was observed and noted as normal if the mouse assumed a wide spread toes and legs position. Cupping of the paws and pulling of the legs in toward the body was noted as the cupping and pulling response [23], [26].

### Ethics Statement

This study was carried out in strict accordance with the Guidelines for the Care and Use of Laboratory Animals of the National Institutes of Health. The protocol was approved by the Indiana University School of Medicine Institutional Animal Care and Use Committee (Protocol Number: 10142). All surgeries were performed under anesthesia, and all efforts were made to minimize animal suffering.

### Rotarod testing

Motor function of WT C57BL/6 (n = 7), Tg-FDD (n = 10), Tg-Tau (n = 11), and Tg-FDD-Tau (n = 9) mice, was tested at six months of age using a rotarod device (Columbus Instruments International, Columbus, OH) as described previously [26]. Only naive mice were used. Each trial lasted a maximum of 10 min, during which time the rotating rod underwent a linear acceleration from 4 to 40 rpm over the first 5 min of the trial and then remained at maximum speed for the remaining 5 min. Animals were scored for their latency to fall (in seconds) for each trial. Animals were rested a minimum of 30 min between trials to avoid fatigue and exhaustion. Each mouse performed four trials on each of four consecutive days.

### Antibodies

For immunohistochemical and biochemical studies polyclonal antibodies (Abs) were raised in rabbits by injecting a synthetic peptide homologous to residues 23–34 (FNLFLNSQEKHYC) of the ADan amyloid peptide [23] (Abs 1699/1700) and a synthetic peptide homologous to residues 42–54 (GLKAEEAGIGDTC) of tau [27] (Ab d29). Commercial polyclonal Abs against glial fibrillary acidic protein (GFAP) (Dako, Carpinteria, CA) for the detection of astrocytes, an Ab against caspase-3, large subunit and proform (AB1899, Millipore, Temecula, CA), and an Ab against BRI_2_ (Itm2b, ab14307, Abcam, Cambridge, MA) were used, as were monoclonal antibodies against synaptophysin (MAB368, Millipore and SY38, Dako), caspase cleaved tau (Tau-C3, MAB5430, Millipore), mouse and human MAPT phosphorylated (p-tau) at Ser202/Thr205 (AT8, Pierce Biotechnology, Rockford, IL), p-tau at Ser212/Thr214 (AT100, Pierce Biotechnology), and beta-actin (AC-15, Sigma, Saint Louis, MO). Anti-keratan sulfate Abs (5D4, Seikagaku Kogyo, Japan) were used for the detection of activiated microglia. For the detection of the Aβ peptide, clone 10D5 (Elan Corporation, San Francisco, CA, USA) and clone 4G8 (SIGNET, Dedham, MA, USA) were used. Secondary Abs used for western blot ECL detection were anti-rabbit IgG, HRP (NA934, GE Healthcare, Piscataway, NJ), anti-mouse IgG, HRP (NA931, GE Healthcare), and anti-chicken IgY, HRP (A9046, Sigma-Aldrich, Saint Louis, MO).

### Histology and immunohistochemistry

Mice were anesthetized and perfusion fixed with 4% paraformaldehyde in 0.1 M phosphate buffer, pH 7.2 (Sigma-Aldrich). Brains were removed, embedded in paraffin, and sectioned. Sections (8 µm thick) were cut and mounted on poly-l-lysine-coated slides and stained with hematoxylin and eosin [23], [26], [27]. Some sections were stained with Bodian silver staining. ThS was used to show the presence of amyloid deposits in the brain [23], [27]. Immunohistochemical staining of mice sections were performed as previously described [23], [27]. Cell counts were performed on four Tg-Tau and four Tg-FDD-Tau mice at 6 and 11 months of age. Individual mice were coded, and for each animal, brains were serially cut in coronal sections (6 µm thick) and immunostained with AT8 and Tau-C3 Abs. The total number of positive neurons throughout the neocortex was estimated on 11 coronal sections at 180 µm intervals following a standard protocol [28]. Nerve cells were counted manually using a 20× or 40× objective. To avoid counting the same neuron in consecutive sections, only neurons with a nucleolus were included. Statistical analysis was done using GraphPad Prism version 5.04 (GraphPad Software, San Diego, CA).

### Protein extraction

Brains were removed, dissected, quickly frozen on dry ice and stored at −80°C. Post-nuclear supernatants (PNS) were prepared by homogenizing the neocortex in 3 volumes of cold Hepes-sucrose buffer (20 mM Hepes, 1 mM EDTA, 1 mM EGTA, 0.25 M sucrose, all from Sigma) containing protease inhibitors (PI) (Complete, 1 mM pepstatin, 100 mM TLCK- HCl, 200 mM TPCK, and 1 mM leupeptin; all from Roche Molecular Biochemical, Indianapolis, IN). The homogenates were centrifuged at 1,000 g for 10 minutes and the supernatants (PNS) retained for western analysis. Soluble and sarkosyl insoluble fractions were prepared from the neocortex as previously described [29]. Tissue was homogenized in 3 volumes of Tris-sucrose buffer (25 mM Tris-HCl, 150 mM NaCl, 1 mM EDTA, 1 mM EGTA, 250 mM sucrose; buffer A) containing PI and phosphatase inhibitors (Phosphatase Inhibitor Cocktail 2, P5726, Sigma). Homogenates were centrifuged for 20 minutes at 15,000 g in a Beckman TLA 110 rotor (Beckman, Palo Alto, CA). The supernatant (soluble fraction) was reserved. The pellet was resuspended in a fresh volume of buffer A and centrifuged 15 minutes at 15,000 g. The supernatant was removed; the pellet was washed with 1 ml buffer A, and centrifuged 15 minutes at 15,000 g. The pellet was resuspended in 2 ml of buffer B (buffer A with 1% sarkosyl), incubated 1 hour at room temperature on a rotator, and centrifuged 30 minutes at 43,000 g at 4°C. The pellet was washed two times with 2 ml of buffer B and centrifuged 30 minutes at 43,000 g at 4°C. The pellet (sarkosyl insoluble fraction) was resuspended in 200 µl of Laemmli sample buffer (Bio-Rad, Philadelphia, PA).

### Western blot analysis

Protein concentrations were determined with the BCA Protein Assay Kit (Pierce). Forty µg of protein from PNS and sarkosyl soluble samples were used for western blot analysis. Five mg tissue w/v (pre-processing tissue weight (mg)/200 µl of sample buffer) of the sarkosyl insoluble fractions were used for western blot analysis. Proteins were separated on Mini-Protean TGX Precast gels (Bio-Rad), run under denaturing conditions and electrotransferred to Immobilon PVDF membrane (Millipore). Membranes were blocked with 0.01% milk in PBS (10 mM phosphate, pH 7.4, 150 mM NaCl) with 0.05% Tween-20 (PBS-T, all from Sigma) using the SNAP ID protein detection system (Millipore) then incubated 2 hours or overnight with the primary Ab. Following washes with PBS-T buffer, membranes were incubated 1 hour with the appropriate HRP-conjugated secondary Ab and visualized using the Pierce ECL Pico kit (Pierce). To ensure equal protein loading of the soluble and PNS samples, blots were probed with anti-β-actin. Blots were scanned and quantified using Image J software from NIH. Statistical analysis was done using GraphPad Prism version 5.04 (GraphPad Software).

### RNA isolation and multiplex expression analysis

For isolation of RNA, three (WT n = 6; Tg-FDD n = 4; Tg-Tau n = 4, and Tg-FDD-Tau n = 6), six (WT n = 6; Tg-FDD n = 5; Tg-Tau n = 5; and Tg-FDD-Tau n = 6), and nine (WT n = 6; and Tg-FDD-Tau n = 6)-month-old mice were anesthetized, perfused transcardially with 0.9% saline, and the brains removed. The hippocampus (HIP) and neocortex (CTX) were dissected, placed in 750 ul of RNA later (Qiagen, Valencia, CA) and frozen at −20 degrees. RNA was isolated from the HIP and CTX samples using RNeasy Lipid Tissue Mini Kit (Qiagen) according to the manufacturer protocol. Samples were treated on column with the RNase free DNase Kit (Qiagen) according to the manufacturer instructions. Reverse transcription was performed on 25 ng of total RNA for each sample followed by multiplex PCR, and fragment separation by capillary electrophoresis using the GeXP Chemistry Protocol (Beckman Coulter, Fullerton, CA). Fragments were separated using a CEQ 8000 Automated Capillary DNA sequencer/Genetic Analysis Systems (Beckman Coulter), and analyzed using the GenomeLab GeXP Genetic Analysis System (Beckman Coulter) using the following fragment analysis parameters: slope threshold = 0.9999, peak height threshold = 500 rfu, peak size<375, peak size>145, dye = D4. Multiplex-specific fragments were selected by applying exclusion filters and the data exported to eXpress Analysis software, where they were normalized against mouse polymerase II polypeptide A (Polr2a) as described [30]. Relative mRNA level values for each of the triplicates for each RNA sample were averaged and the mean for the replicates were compared by unpaired two-tailed t-tests using GraphPad Prism version 5.04 (GraphPad Software). Differences in relative mRNA levels with p-values<0.05 were considered statistically significant.

## Results

### Generation and behavioral analysis of Tg-FDD-Tau mice

**Figure 1:**
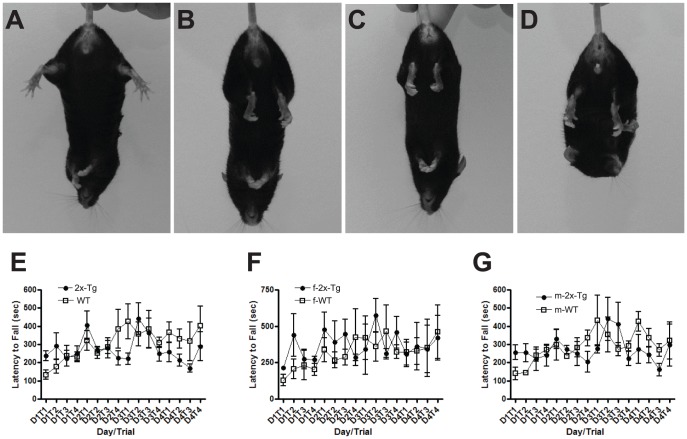
Comparison between single transgenic mice, double transgenic mice, and littermate controls. Representative photographs of 12 months old wild-type (A), Tg-Tau (B), Tg-FDD-Tau (C), and Tg-FDD (D) showing clasping of the hindlimb and bilaterally pulling of the hind paws toward the abdomen when suspended by the tail. Performance of wild-type (WT, n = 7) and Tg-FDD-Tau (2x-Tg, n = 9) animals on an accelerating rotating rod apparatus at 6 months of age (E). No significant differences in performance were observed between Tg-FDD-Tau mice compared with age-matched WT animals. No differences in daily performances were observed in females (f) (F) and males (m) (G) WT and Tg-FDD-Tau animals.

Compared to WT mice, male and female Tg-FDD-Tau mice exhibited significant weight loss by 6 months of age (female, p = 0.0181; male, p = 0.0145); however, the weight of Tg-FDD-Tau mice was not significantly different from that of single Tg-FDD and Tg-Tau mice of the same age. Tg-FDD-Tau, Tg-FDD, and Tg-Tau mice exhibited abnormal grooming behavior starting around 9 months as apparent by their dull rough coats. By 12 months of age, Tg-FDD-Tau mice showed a hunched back or arched posture when sitting and walking, which could also be observed in some Tg-FDD mice. None of these abnormalities were seen in WT mice of the same age. Twelve month old Tg-FDD-Tau mice and Tg-FDD mice displayed an unusual gait in which the mouse holds its body near the walking surface and takes wide shortened steps, which was not observed in WT mice and Tg-Tau mice of the same age. At 12 months of age, 88% (n = 8) of Tg-FDD-Tau mice, 33% (n = 6) of the age-matched WT mice, 100% of Tg-Tau (n = 5), and 77% (n = 9) of Tg-FDD exhibited cupping of the hind paws and bilaterally pulling of the hind paws toward the abdomen when suspended by the tail (Fig. 1A–D). Beyond 12 months of age, the health of Tg-FDD-Tau mice, like that of single Tg-Tau mice, sharply declined and the mice had to be euthanized in accordance with institutional guidelines. At 10–14 months, Tg-Tau mice showed a precipitous decline in health, with deterioration of coat condition, hunched back, weight loss, and hind-limb dystonia. No significant differences were observed in the motor function between single Tg-Tau and single Tg-FDD mice (not shown) and Tg-FDD-Tau mice compared with WT mice at 6 months of age by rotarod analysis when male and female animals were analyzed together or when females and males were analyzed separately (Fig. 1E–G). Additional rotarod analysis in older mice was not performed because the decline in motor performance that led to a reduced survival rate in Tg-Tau mice beginning around 9 months of age was also observed in Tg-FDD-Tau mice.

### Transgene expression in Tg-FDD-Tau mice

**Figure 2:**
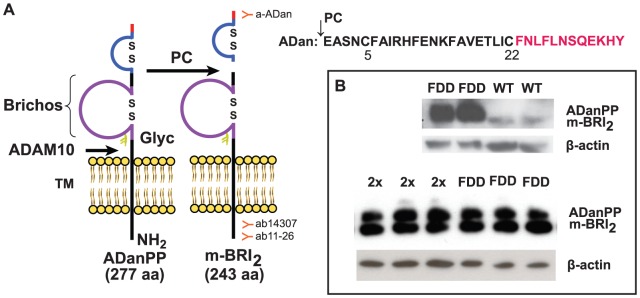
BRI_2_ expression and processing in transgenic mice. Schematic diagram of the Danish amyloid precursor protein (ADanPP) (A). BRI_2_ is a type-II single trans-membrane (TM) domain protein. Processing of ADanPP by pro-protein convertases (PCs) generates the 34 amino acid peptide (ADan) and a mature form of BRI_2_ (m-BRI_2_). Processing by ADAM10 in the ectodomain of BRI_2_ releases the BRICHOS domain and an N-terminal fragment (NTF). The NTF is also the subject of additional proteolysis by SPPL2, releasing an intracellular domain (ICD) and a C-terminal peptide fragment (BRI2 C-peptide) [18]. Disulfide bonded loops in the BRICHOS domain and in the carboxy-terminus of BRI_2_ (amino acids 5 and 22 of the ADan peptide) are indicated. The figure shows the localization of the Abs used. Western blot analysis of PNS from neocortex of Tg-FDD-Tau (2x, n = 6) mice, Tg-FDD (FDD, n-6) mice, and wild-type (WT, n = 6) control mice using the BRI_2_-amino-terminal antibody 14307 (B). The ectodomain processing of the FDD mutant form of BRI_2_ by PCs seems to be compromised in FDD. In Tg-FDD mice, two bands can be observed corresponding to full-length ADanPP and m-BRI_2_, while in WT mice most of the detectable BRI_2_ protein can be seen as m-BRI_2_. Samples were run in triplicates. Representative samples are shown. The densitometric values of the bands representing ADanPP and m-BRI_2_ immunoreactivity were normalized to the values of the corresponding actin band using ImageJ software. No significant differences were observed between Tg-FDD-Tau and Tg-FDD mice (independent *t* test).

Cleavage of the FDD mutant form of BRI_2_ (ADanPP) between amino acids Arg243 and Glu244 by PCs releases the 34 amino acids ADan peptide (Fig. 2A) and a mature form of BRI_2_ (m-BRI_2_) [9], [18]. As previously reported for transfected cells and FDD knock-in mice [31], the ectodomain processing of the FDD mutant form of BRI_2_ by PCs seems to be compromised in FDD, with an accumulation of full-length ADanPP as immature BRI_2_ mutant protein (Fig. 2B). No significant difference was observed in the expression of ADanPP between Tg-FDD and Tg-FDD-Tau mice (Fig. 2B). Densitometric scanning and statistical analysis of the bands indicate that the levels of ADanPP and m-BRI_2_ were not statistically different in Tg-FDD-Tau and in Tg-FDD mice. We observed that the higher molecular weight band, representing immature BRI_2_, and the lower molecular weight band, representing m-BRI_2_, had approximately the same intensity, suggesting that ∼50% of the immature protein was being processed to m-BRI_2_ protein in Tg-FDD-Tau and Tg-FDD mouse lines.

The integration and expression of the tau transgene was analyzed in Tg-Tau mice. Southern blot analyses of Tg-Tau mice showed one integration site and incorporation of ∼60–70 copies of the transgene (not shown). Males only carried one copy of the transgene since transgene integration occurred on the murine X chromosome. Analysis of the expression of the tau transgene between single Tg-Tau and Tg-FDD-Tau mice by multiplex PCR did not reveal any significant differences in tau expression between the two lines (Fig. S1).

### Pathological analysis of Tg-FDD-Tau mice

**Figure 3:**
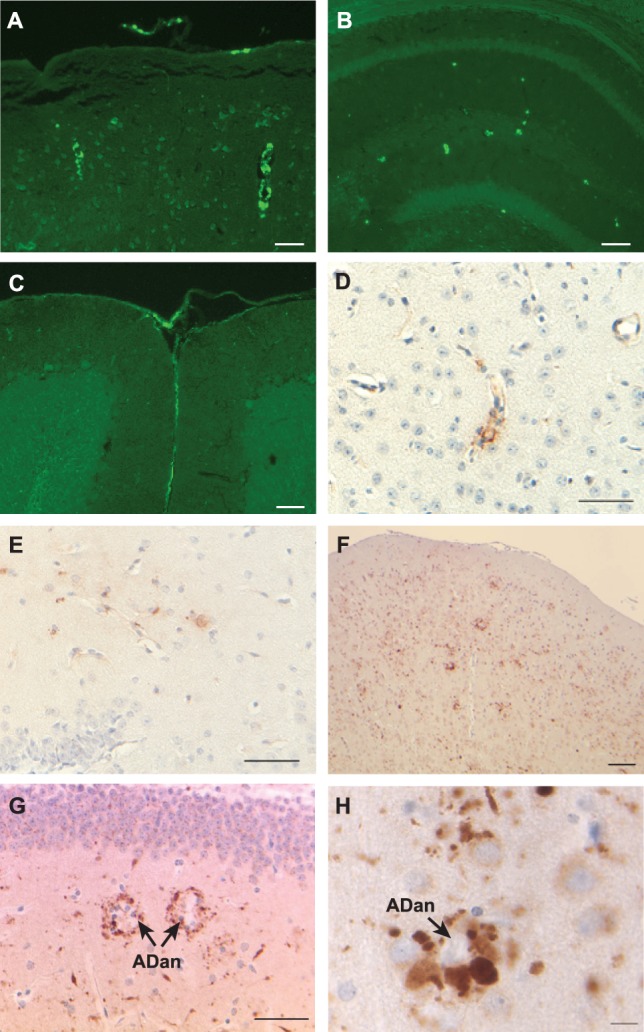
Amyloid deposition and BRI_2_ accumulation in transgenic mice. Amyloid deposition is seen throughout all cortical layers (A), the hippocampal formation (B), and in leptomeningeal vessels of the cerebellum (C) of Tg-FDD-Tau mice. Antibodies against ADan immunolabeled cortical blood vessels (D) and amyloid deposits in the hippocampus (E). Amyloid plaques in Tg-FDD mice are surrounded by globular dystrophic neurites (DNs) labeled by the BRI_2_-amino-terminal Ab 14307 in the neocortex (F) and hippocampus (G, H). The Ab also labeled intracellular deposits and swollen neurites. Arrows indicate the presence of ADan amyloid plaques. Sections were from a 12 month old (A–E) Tg-FDD-Tau mice and a 21 month old Tg-FDD mouse (F–H). Thioflavine S (A–C). Immunohistochemistry using Abs 1699/1700 (D, E) and Ab 14307 (F–H). Scale bars: A–C, F, 100 µm; G, 50 µm; H, 25 µm.

**Figure 4:**
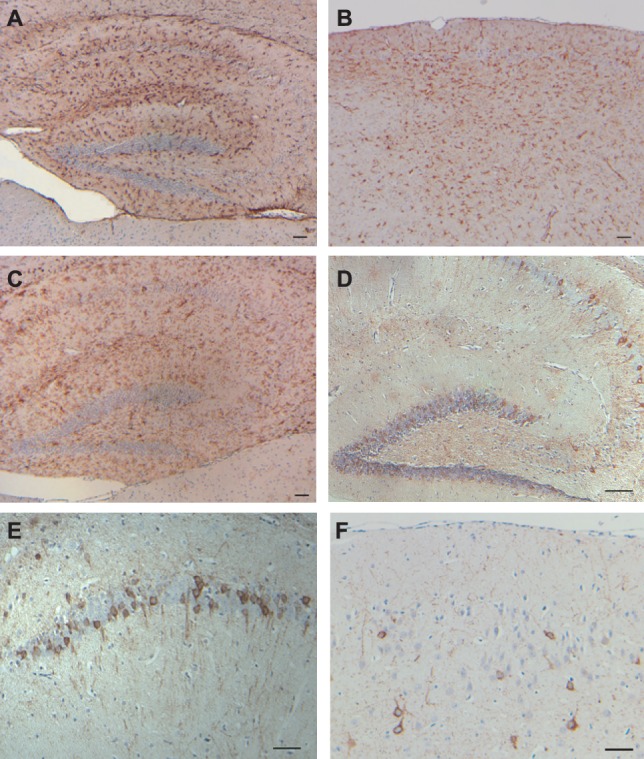
Tau deposition and inflammatory changes in double transgenic mice. Reactive astrocytes in the hippocampus (A) and neocortex (B), and keratan sulfate-positive-activated microglia in the hippocampus (C). The phosphorylation-dependent anti-tau Ab AT8 immunolabeled tau deposits in the hippocampus (D, E), and the neocortex (F). Sections were from 6 (F), 10 (B), and 12 month old (A, C, D, E) Tg-FDD-Tau mice. Immunohistochemistry using anti-GFAP (A, B), anti-keratan sulfate (C), and Ab AT8 (D–F). Scale bars: A–F, 50 µm.

A similar progression and distribution of ADan deposition was observed between the Tg-FDD-Tau and the Tg-FDD lines [23]. Beginning at around 6–7 months of age, Tg-FDD-Tau mice showed amyloid deposition primarily in leptomeningeal cerebellar vessels, developing later extensive amyloid lesions in the parenchyma and vasculature of the neocortex, hippocampus, and cerebellum (Fig. 3A–C). Parenchymal amyloid deposition in the hippocampus was most prominent in the CA3 and CA2 regions and the hilus, consistent with the neuropathological findings in Tg-FDD mice [23]. Anti-ADan Abs immunolabeled amyloid plaques, diffuse ADan deposits, and vascular deposits in Tg-FDD-Tau mice (Fig. 3D,E). Immunohistochemistry using anti-ADan Abs (which also recognize the C-terminus of ADanPP) in Tg-FDD mice revealed the presence of parenchymal and vascular amyloid deposits, as well as intracellular deposits [23]. By using Abs against the N-terminus of BRI_2_ (Fig. 2A) we observed the presence of clusters of swollen neuritic profiles at the periphery of ADan plaques, in addition to intracellular BRI_2_ deposits (Fig. 3F–H). DNs were made up of large and rounded processes, strongly immunolabelled with the Abs against the N-terminus of BRI_2_ but were not recognized by anti-ADan Abs. These profiles appear to be associated with fibrillar ADan amyloid cores, although some clusters of DNs were also observed in the absence of ADan deposits (Fig. 3G,H). Similar immunoreactive profiles were observed using the BRI_2_-N-terminal Ab BRI-11-26 (not shown) [32]. No immunoreactivity was seen when the primary Ab was omitted. Increased expression of glial fibrillary acidic protein (Gfap), which occurs during activation of astrocytes, was observed by immunohistochemistry (Fig. 4A,B). In addition, 5D4-immunopositive microglia was observed throughout neocortical areas and the hippocampus of Tg-FDD-Tau mice (Fig. 4C). These immunohistochemical observations were confirmed by gene expression analysis. A significant increase in the expression levels of *Gfap* and the microglial-specific ionized calcium binding adapter molecule 1 (*Iba1*) was observed in Tg-FDD-Tau mice compared to WT mice (Fig. S2). We also observed an increase in the expression of the complement protein *C1q*, and the pro-inflammatory chemokines macrophage inflammatory protein-1α (*Ccl3*) and *Ccl5/RANTES* (Fig. S2). The distribution of tau deposits was compared between single Tg-Tau and Tg-FDD-Tau mice. Tau deposits in Tg-FDD-Tau mice were found predominantly in the hippocampus, piriform cortex, brain stem, spinal cord, and the cerebellum (Fig. 4D–F), as in single Tg-Tau mice (Fig. S3) [24]. Tau accumulation was detected with the phosphorylation-dependent tau Ab AT8 and was never found in control animals. In FDD, amyloid-β (Aβ) may co-deposit with ADan, mainly in vascular and perivascular amyloid lesions [4], [14]. However, immunohistochemical analysis using Abs against Aβ did not show any immunopositivity in brain sections from single Tg-FDD and Tg-FDD-Tau mice (not shown).

### Enhanced tau pathology and cleavage of tau at D421 in Tg-FDD-Tau mice

**Figure 5:**
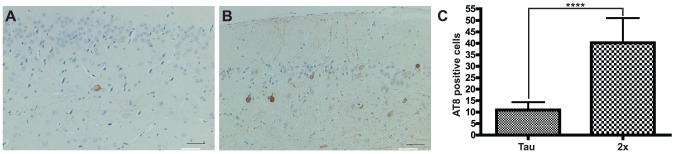
Enhanced AT8 immunopositive neuronal perikarya in Tg-FDD-Tau mice. Staining of a section of the neocortex of a 6 month old Tg-Tau mouse (A) and a Tg-FDD-Tau (B) mouse. A statistically significant difference in the number of AT8-positive neuronal perikarya is observed between Tg-FDD-Tau (2x) mice and single Tg-Tau (Tau) mice (C) (****P<0.0001, two tail *t* test). Immunohistochemistry using Ab AT8 (A, B). Scale bars: A, B, 50 µm.

**Figure 6:**
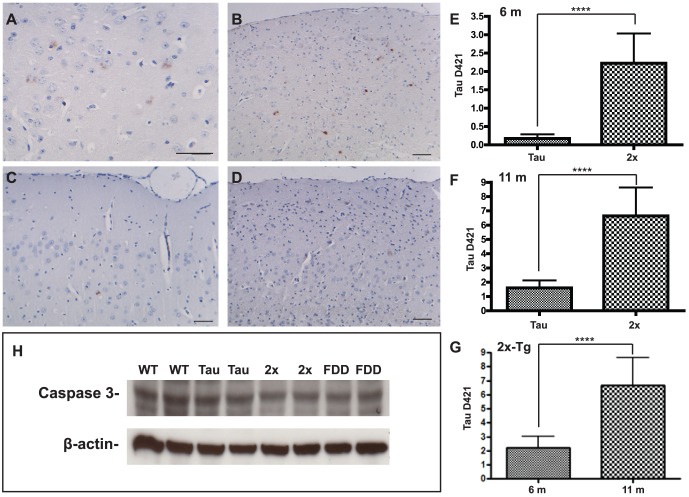
Enhanced Tau-C3-positive neuronal perikarya in Tg-FDD-Tau mice. Analysis of caspase-truncated tau at D421. Serial sections of the neocortex of Tg-FDD-Tau (A, B) and single Tg-Tau (C, D) mice were analyzed at 6 (A, C) and 11 (B, D) months of age. A statistically significant difference in the number of Tau-C3-positive neuronal perikarya is observed between Tg-FDD-Tau (2x) mice and single Tg-Tau (Tau) mice at 6 (E) and 11 (F) months of age. The number of Tau-C3-positive neuronal perikarya in Tg-FDD-Tau mice increased with statistical significance between 6 and 11 months of age (G). (****P<0.0001, two tail *t* test). Representative immunoblots showing pro-caspase-3 levels in PNS from 11 months old wild-type (WT, n = 6), Tg-Tau (Tau, n = 6), Tg-FDD-Tau (2x, n = 6), and Tg-FDD (FDD, n = 6) mice (H). Samples were run in triplicates. No significant differences in the levels of pro-caspase-3 was observed between the four groups. β-actin was used to normalize protein loading. Optical densities of the individual bands were quantified using NIH ImageJ. Statistical analyses were performed with GraphPad Prism 5.04. Immunohistochemistry using Ab Tau-C3 (A–D). Scale bars: A–D, 50 µm.

**Figure 7:**
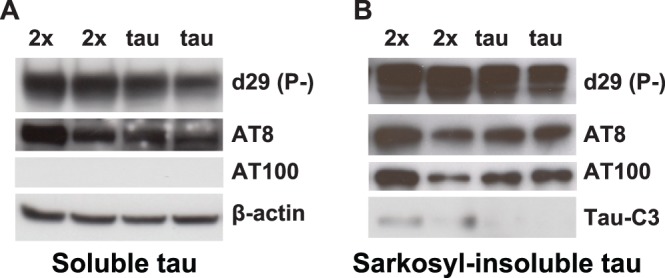
Biochemical analysis of tau in transgenic mice. Representative immunoblots of tris-soluble and sarkosyl-insoluble fractions from 9 month old Tg-FDD-Tau (2x) and Tg-Tau (Tau) (four to five mice per group were analyzed). Samples were run on SDS-PAGE and immunoblotted with anti-tau Abs d29, AT8, AT100 and Tau-C3. β-actin was used to normalize protein loading.

Extracellular amyloid deposition in combination with NFT formation may be seen only in a limited number of human neurodegenerative diseases, including FDD [3], [4], [15]–[18]. To assess the influence of the expression of the Danish mutant form of BRI_2_ on tau pathology, we analyzed AT8 detectable p-tau in neuronal cell bodies of Tg-Tau and Tg-FDD-Tau mice (Fig. 5A,B). At 6 months of age, we observed by immunohistochemistry a statistically significant difference in the number of AT8-positive (p<0.0001) neuronal perikarya between Tg-FDD-Tau mice and single Tg-Tau mice (Fig. 5C). This change occurred at an age before any significant ADan deposition can be observed in Tg-FDD-Tau mice. Since truncation of tau has been proposed to cause filament formation [19]–[22], the presence of caspase-truncated tau at Asp 421 was assessed using Ab Tau-C3. Immunohistochemical studies showed a significant increase in the number of Tau-C3-positive neuronal perikarya in Tg-FDD-Tau mice compared to single Tg-Tau mice at 6 months of age (Fig. 6A,C,E) and at 11 months of age (Fig. 6B,D,F). The number of Tau-C3-positive neuronal perikarya in Tg-FDD-Tau mice increased progressively with age, as it was also statistically significant between 6 and 11 months of age (Fig. 6A,B,G). Analysis of pro-caspase-3 levels by immunoblot showed no significant differences between WT, Tg-Tau, Tg-FDD-Tau and Tg-FDD mice (Fig. 6H). Soluble and sarkosyl-insoluble tau prepared from the brain of Tg-Tau and Tg-FDD-Tau mice were analyzed by western blot. In the soluble brain fraction, we observed strong immunoreactivity using phosphorylation-independent (d29) and phosphorylation-dependent (AT8) Abs, but not using the phosphorylation-dependent Ab AT100 (Fig. 7A) and the Ab specific for tau truncated at Asp 421 (Tau-C3, not shown). Sarkosyl-insoluble tau was detected using the phosphorylation-independent Ab d29, and the phosphorylation-dependent Abs AT8 and AT100 (Fig. 7B). A faint immunoreactive band corresponding to tau protein truncated at Asp 421 was observed only in the sarkosyl-insoluble fraction of Tg-FDD-Tau mice using Ab Tau-C3.

### Decreased levels of synaptophysin in Tg-FDD-Tau mice precede ADan plaque formation and significant tau deposition

**Figure 8:**
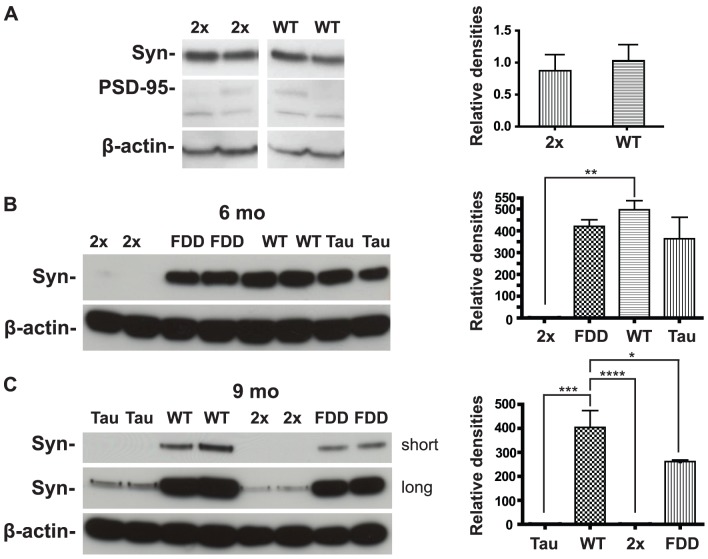
Biochemical analysis of synaptophysin levels in transgenic mice. Representative immunoblots and densitometry analysis showing synaptophysin and PSD-95 levels in PNS from wild-type (WT, n = 6), Tg-FDD (FDD, n = 6), Tg-Tau (Tau, n = 6), and Tg-FDD-Tau (2x, n = 6) mice. Samples were run in triplicates. No significant changes were observed at 3 months of age in synaptophysin levels between Tg-FDD-Tau and WT mice (A). A significant decrease in synaptophysin levels in Tg-FDD-Tau mice is observed at 6 months of age (B). At 9 months of age, synaptophysin levels are also decreased in single transgenic mice, particularly in Tg-Tau mice (C). A short and an extended (long) exposure of the film are shown. β-actin was used to normalize protein loading. Optical densities of the individual bands were quantified using NIH ImageJ. Statistical analyses were performed with GraphPad Prism 5.04. (*P<0.05; **P<0.01; ***P<0.001; ****P<0.0001, two tail t test).

Losses of the pre-synaptic vesicle protein synaptophysin have been shown to correlate with cognitive decline in AD cases [33], [34]. Several groups have shown a decrease in dendritic spine density and synaptophysin-positive synapses radiating out from the surface of plaques in mouse models [35]–[38]. To assess the effect of tau and ADan on synapses, we performed biochemical analysis by Western blot on PNS-soluble brain fractions. The relative amount of protein was quantified by densitometry and normalized with actin. No significant differences in the levels of synaptophysin and the post-synaptic density protein 95 (PSD-95) were observed at the young ages tested, 2 (not shown) and 3 months between control mice and Tg-FDD-Tau mice (Fig. 8A). Remarkably, we observed a clear decrease in synaptophysin levels in Tg-FDD-Tau mice (p = 0.005) at 6 months of age (Fig. 8B). Synaptophysin levels were found to be slightly decreased in single transgenic mice at 6 months of age, but the decrease did not reach statistical significance (Fig. 8B). Synaptophysin immunostaining did not show a remarkable difference in immunostaining between controls and single FDD and Tau transgenic mice in hippocampal CA1. A decrease in immunostaining was observed only in Tg-FDD-Tau mice (Fig. S4). Quantitative studies using stereological techniques will be needed to evaluate synaptic pathology in Tg-FDD-Tau mice. In older animals (9 months of age), synaptophysin levels were found to be decreased in all transgenic mice lines. The decrease was particularly significant in single Tg-Tau mice, but it was also statistically significant in single Tg-FDD mice (Fig. 8C). No significant difference in synaptophysin levels was observed between single Tg-Tau and Tg-FDD-Tau mice at 9 months of age; however, the difference in synaptophysin levels between single transgenic mice (Tg-FDD and Tg-Tau) and between Tg-FDD and Tg-FDD-Tau reached statistical significance.

## Discussion

In the present study, we have characterized a double transgenic mouse model that over-expresses the FDD-mutant form of BRI_2_ (ADanPP) and the smallest 4-repeat isoform of mutant tau (P301S), with both transgenes being driven by the murine *Prnp* promoter. The Tg-FDD-Tau model was designed to reproduce the neuropathologic features of FDD and to test, *in vivo*, the ability of ADan to modify tau pathology. Tg-FDD-Tau mice displayed clasping and limb retraction when lifted by the tail, revealing a motor impairment that is age-dependent. On immunohistochemical examination, Tg-FDD-Tau mice exhibited an age-dependent accumulation of both ADan and tau; however, the results of this study show that expression of human mutant BRI_2_ enhances tau phosphorylation and C-terminal cleavage of neuronal tau, while ADan deposition remained comparable to its parental strain. Enhancement of tau accumulation in Tg-FDD-Tau mice occurs before extracellular deposition of ADan can be detected; suggesting that soluble ADan species or abnormal function of mutant BRI_2_ may mediate tau pathology. Sarkosyl-insoluble tau extracted from both Tg-FDD-Tau and Tg-Tau mice contained a major hyperphosphorylated species migrating at 64 kD. Although the levels of sarkosyl-insoluble tau extracted from Tg-FDD-Tau mice were elevated compared to Tg-Tau mice, the change did not reach statistical significance at the age analyzed. These data is in agreement with previous observations of enhanced tau pathology in mutant tau-expressing mice injected with Aβ_42_ peptides [39] or co-expressing mutant *AβPP* or *BRI_2_* in their brains [40]–[42], supporting the hypothesis that amyloidosis facilitates tauopathy in AD. However, the exact mechanism(s) involved in promoting accelerated neurofibrillary degeneration in double amyloid/tau mouse models remains to be determined. Tau truncation by caspases has been proposed as an early and crucial step in the development and maturation of NFTs [43]. Caspase activation may precede and lead to tangle formation with tangle-bearing neurons representing survivors of caspase attack [22]. By immunohistochemistry, we detected intracellular accumulation of tau truncated at Asp 421 in single Tg-Tau and Tg-FDD-Tau mice. The number of Tau-C3 positive cells was significantly higher in Tg-FDD-Tau mice compared to single Tg-Tau mice; however, the number of Tau-C3 positive cells represented only a small fraction of the number of neurons that were labeled by using Ab AT8 on the same section in both mouse lines. Truncation of tau at Asp 421 was not detected by western blot using the Ab Tau-C3 in single Tg-Tau mice, as previously reported on P301S transgenic mice [21], [44]. Low levels of insoluble truncated tau were observed only in the sarkosyl-insoluble fraction of Tg-FDD-Tau mice brains, which may reflect the higher number of cells that were detected using Ab Tau-C3 by immunohistochemistry in Tg-FDD-Tau mice compared to Tg-Tau mice. Although tau truncation appears to play an important role in the assembly of tau into filaments, it is still controversial whether tau truncation is primary or secondary to tau hyperphosphorylation. Our data, in agreement with previous reports on single Tau transgenic and double transgenic models expressing mutant tau and amyloid precursor protein [19]–[22], [44], strongly suggest that ADan and Aβ may act on a similar cellular degenerative pathway, marked by caspase activation and tau fibrillar deposition and neurodegeneration [19], [45].

One prominent finding in FDD as well as in AD and non-AD tauopathies [14], [46], [47] is the presence of a neuroinflammative process. Immunohistochemical and gene expression studies showed clear evidence of neuroinflammation in Tg-FDD-Tau mice. Activated astrocytes and microglia were seen frequently present in close proximity of amyloid deposits and NFTs. Interestingly, activated microglia was detected even before ADan deposition was observed and correlated with tau deposition in both Tg-Tau and Tg-FDD-Tau mice. The finding of an inflammatory process in association with tau deposition is in agreement with previous work done in transgenic mouse models expressing mutant tau protein and may constitute a general response of neurons to the accumulation of aggregated tau [46]–[49]. We observed that ADan amyloid cores in Tg-FDD mice are surrounded by DNs consisting of large globular processes that were labeled by Abs against the N-terminus of BRI_2_. The lack of immunoreactivity of DNs using a C-terminal Ab against ADan in Tg-FDD mice suggests that the accumulation of BRI_2_ involves a form of BRI_2_ generated after PC processing. This BRI_2_ accumulation may be associated with disruption of axoplasmic transport by the presence of abnormal tau near amyloid deposits [34], [50], [51]. These globular processes have been shown to contain large numbers of clustered vesicles that can be labeled by Abs against pre-synaptic proteins [35], [52]. Further studies will determine whether the large dystrophic processes identified by BRI_2_ immunostaining also overlap with AβPP and ubiquitin immunoreactivity, and whether they play a role in neurodegeneration.

In AD, the relationship among amyloid deposition, NFT formation, and synaptic loss remains obscure [33]–[36]. Early loss of synaptic contacts has been associated with the initiation of cognitive decline, followed by neuritic damage, and neuronal cell loss [53], with loss of hippocampal synaptophysin correlating with cognitive decline in AD [34]. Synaptic and cognitive dysfunction have been documented in several transgenic models of AD before amyloid plaque formation occurs [54], [55], suggesting that oligomeric forms of Aβ may play a role in synapse loss in AD [56]. To characterize changes in synaptic integrity in Tg-FDD-Tau mice, we measured levels of the pre-synaptic marker synaptophysin in brains of WT, Tg-FDD, Tg-Tau and Tg-FDD-Tau mice. We observed that at 6 months of age, brain synaptophysin levels were significantly decreased in Tg-FDD-Tau mice compared with control and single transgenic mice, while synaptophysin levels of Tg-FDD and Tg-Tau mice were not significantly different from those of age-matched non-transgenic controls. As animal aged, synaptophysin levels of single Tg-FDD and Tg-Tau mice also decreased significantly from those of age-matched non-transgenic controls. Together, these results show an age-dependent synaptic degeneration occurring in single Danish and tau transgenic mice that is particularly significant in double Tg-FDD-Tau mice, where the presence of the Danish mutant form of BRI_2_ and the P301S mutant form of tau lead to an earlier and more severe loss of synaptophysin. The loss of synaptophysin in Tg-FDD-Tau mice occurs before amyloid plaques can be detected and significant tau pathology is present. Oligomeric forms of ADan, which were described in single Tg-FDD mice [23], may directly or indirectly interact with mutant tau leading to a reduction of tau binding to microtubules and consequent impairment in transport [48], and perhaps to BRI_2_ accumulation in DNs. Several studies using animal models have shown that tau plays an important role in synaptic loss and synaptic dysfunction, with synaptic dysfunction appearing before the formation of NFTs, possibly involving oligomeric forms of tau [43], [57]. In addition, Tg-FDD and Tg-FDD-Tau mice show an abnormal processing pattern of BRI_2_, with accumulation of immature mutant BRI_2_. Abnormal processing of BRI_2_ can lead to a decrease in m-BRI_2_ levels and accumulation of murine amyloid β precursor protein (Aβpp) C-terminal fragments [58], which together with mutant tau may cause synaptic dysfunction in Tg-FDD-Tau mice, independent of the presence of ADan oligomers. Work done using knock-in mice with the Danish mutation (FDD-KI) and the British mutation (FBD-KI) in the murine *Bri_2_* gene, and with mice with a knock-out of one allele of murine *Bri_2_* (*Bri_2_^+/−^*) has shown that these mice exhibit abnormal synaptic plasticity and memory deficits that is independent of the presence of the amyloid peptides [31], [59]–[61]. The finding of a reduction of m-Bri_2_ in synaptic membranes of FDD^KI/+^ mice in the absence of ADan and tau deposition is consistent with the hypothesis that FDD may begin as a synaptic disease, associated with a Bri_2_ loss of function [31].

In summary, this study has shown the interrelationship between ADan (and BRI_2_) and cytoskeletal changes that may be at play in FDD. Importantly, we observed enhanced tauopathy, tau truncation, and synaptic loss prior to ADan deposition in the brain of Tg-FDD-Tau mice. This model recapitulates major pathologic characteristics of FDD and may be particularly useful to study early stages of FDD by providing evidence supporting a role for plaque-independent amyloid toxicity in the pathogenesis of FDD.

## Supporting Information

Figure S1 Multiplex RT-PCR expression analysis of tau transgene expression. Bar graphs depict differential tau transgene expression levels between Tg-Tau (Tau, n = 5) and Tg-FDD-Tau (2x, n = 6) mice at 6 months of age. Multiplex RT-PCR analysis was performed on mRNA isolated from the hippocampus (A) and from the neocortex (B). Analysis was performed in triplicate and normalized to the *Polr2a* gene. Group averages are reported as relative mRNA levels (means±sem). No significant expression differences were found by 2-tailed *t* test.
(EPS)

Figure S2 Analysis of inflammatory changes by multiplex RT-PCR expression analysis. Bar graphs depict relative expression levels in wild-type (WT, n = 6) and Tg-FDD-Tau (2x, n = 6) mice at 9 months of age. A significant increase in the expression levels of GFAP in Tg-FDD-Tau mice compared to WT mice is observed in the hippocampus (A) and in the neocortex (B). The expression of the complement protein C1q (C), the proinflammatory chemokines macrophage inflammatory protein-1α (Mip1-α or Ccl3) (D) and Ccl5/RANTES (E), and the microglial-specific ionized calcium binding adapter molecule 1 (Iba1) (F) were also significantly increased in Tg-FDD-Tau mice compared to WT mice. Multiplex RT-PCR analysis was performed on mRNA isolated from the hippocampus (A, C–F) and from the neocortex (B). Analysis was performed in triplicate and normalized to the *Polr2a* gene. Group averages are reported as relative mRNA levels (means±sem). Significant expression differences were found by 2-tailed *t* test.
(EPS)

Figure S3 Neuropathologic examination of transgenic mice expressing the P301S mutation (Tg-Tau). ThS-fluorescent (A) and argentophilic (B) deposits in neurons of the frontal and temporal lobes, hippocampus, piriform cortex, brain stem and spinal cord. Neurons of the temporal cortex show argentophilia in the perikaryon extending into cell processes (B). Phosphorylation-dependent anti-tau Abs showed the presence of tau-immunopositive deposits in both nerve (C, neurons of the frontal cortex) and glial (D, oligodendroglial cells of the white matter; E, astrocytes) cells in several areas of the central nervous system with deposition beginning in the temporal lobe, amygdala and hypothalamus. At the age of 5–14 months, tau positive inclusions were observed in the cingulate, somatosensory, motor, and entorhinal cortices as well as the hippocampus, caudate nucleus, putamen, cerebellum, midbrain, pons, medulla, and the anterior and posterior gray horns of the spinal cord (F, hippocampus; G, temporal cortex). Neuronal loss was observed in the temporal lobe, amygdala and hippocampal pyramidal layer. Sections were from a 10 month old homozygous female (A, B), a 12 month old male (C–E), and a 14 month old male (F, G). Immunohistochemistry using Ab AT8 (C–G). Original magnifications 25× (A, F), 40× (B, G), 63× (C–E).
(TIF)

Figure S4 Neuropathologic analysis of synaptophysin in transgenic mice. Representative microphotographs of synaptophysin immunostaining in the hippocampal CA1 region of 6 months old wild-type (A, B), Tg-FDD (C, D), Tg-Tau (E, F), and Tg-FDD-Tau (G, H) mice. Two mice of each genotype are shown. Immunohistochemistry using Ab SY38. Scale bars: A–H, 50 µm.
(TIF)

## References

[1] Worster-Drought C, Hill TR, McMenemey WH (1933) Familial presenile dementia with spastic paralysis. J Neurol Psychopathol 14: 27–34. 21610757 10.1136/jnnp.s1-14.53.27 PMC1038860

[2] Strömgren E, Dalby A, Dalby MA, Ranheim B (1970) Cataract, deafness, cerebellar ataxia, psychosis and dementia- a new syndrome. Acta Neurol Scand 46 (S43) 261. 10.1111/j.1600-0404.1970.tb02219.x 5457846

[3] Vidal R, Frangione B, Rostagno A, Mead S, Revesz T, et al (1999) A stop-codon mutation in the BRI gene associated with familial British dementia. Nature 399: 776–781. 10391242 10.1038/21637

[4] Vidal R, Revesz T, Rostagno A, Kim E, Holton JL, et al (2000) A decamer duplication in the 3′ region of the BRI gene originates an amyloid peptide that is associated with dementia in a Danish kindred. Proc Natl Acad Sci USA 97: 4920–4925. 10781099 10.1073/pnas.080076097 PMC18333

[5] Vidal R, Delisle MB, Ghetti B (2004) Neurodegeneration caused by proteins with an aberrant carboxyl-terminus. J Neuropathol Exp Neurol 63: 787–800. 15330334 10.1093/jnen/63.8.787

[6] Vidal R, Calero M, Revesz T, Plant G, Ghiso J, et al (2001) Sequence, genomic structure and tissue expression of Human BRI_3_, a member of the BRI gene family. Gene 266: 95–102. 11290423 10.1016/s0378-1119(01)00374-2

[7] Kim SH, Wang R, Gordon DJ, Bass J, Steiner DF, et al (1999) Furin mediates enhanced production of fibrillogenic ABri peptides in familial British dementia. Nat Neurosci 2: 984–988. 10526337 10.1038/14783

[8] Kim SH, Creemers JW, Chu S, Thinakaran G, Sisodia SS (2002) Proteolytic processing of familial British dementia-associated BRI variants: evidence for enhanced intracellular accumulation of amyloidogenic peptides. J Biol Chem 277: 1872–1877. 11709554 10.1074/jbc.M108739200

[9] Choi SI, Vidal R, Frangione B, Levy E (2004) Axonal transport of British and Danish amyloid peptides via secretory vesicles. FASEB J 18: 373–375. 14656991 10.1096/fj.03-0730fje

[10] Sanchez-Pulido L, Devos D, Valencia A (2002) BRICHOS: a conserved domain in proteins associated with dementia, respiratory distress and cancer. Trends Biochem Sci 27: 329–332. 12114016 10.1016/s0968-0004(02)02134-5

[11] Martin L, Fluhrer R, Reiss K, Kremmer E, Saftig P, et al (2008) Regulated intramembrane proteolysis of Bri2 (Itm2b) by ADAM10 and SPPL2a/SPPL2b. J Biol Chem 283: 1644–1652. 17965014 10.1074/jbc.M706661200

[12] Martin L, Fluhrer R, Haass C (2009) Substrate requirements for SPPL2b-dependent regulated intramembrane proteolysis. J Biol Chem 284: 5662–5670. 19114711 10.1074/jbc.M807485200

[13] Holton JL, Ghiso J, Lashley T, Rostagno A, Guerin CJ, et al (2001) Regional distribution of amyloid-Bri deposition and its association with neurofibrillary degeneration in familial British dementia. Am J Pathol 158: 515–526. 11159188 10.1016/S0002-9440(10)63993-4 PMC1850296

[14] Holton JL, Lashley T, Ghiso J, Braendgaard H, Vidal R, et al (2002) Familial Danish dementia: a novel form of cerebral amyloidosis associated with deposition of both amyloid-Dan and amyloid-beta. J Neuropathol Exp Neurol 61: 254–267. 11895040 10.1093/jnen/61.3.254

[15] Ghetti B, Tagliavini F, Masters CL, Beyreuther K, Giaccone G, et al (1989) Gerstmann-Sträussler-Scheinker disease. II. Neurofibrillary tangles and plaques with PrP-amyloid coexist in an affected family. Neurology 39: 1453–1461. 2573006 10.1212/wnl.39.11.1453

[16] Giaccone G, Tagliavini F, Verga L, Frangione B, Farlow MR, et al (1990) Neurofibrillary tangles of the Indiana kindred of Gerstmann-Sträussler-Scheinker disease share antigenic determinants with those of Alzheimer disease. Brain Res 530: 325–329. 2176119 10.1016/0006-8993(90)91304-y

[17] Giaccone G, Mangieri M, Capobianco R, Limido L, Hauw JJ, et al (2008) Tauopathy in human and experimental variant Creutzfeldt-Jakob disease. Neurobiol Aging 29: 1864–1873. 17560687 10.1016/j.neurobiolaging.2007.04.026

[18] Garringer HJ, Murrell J, D'Adamio L, Ghetti B, Vidal R (2010) Modeling familial British and Danish dementia. Brain Struct Funct 214: 235–244. 19779737 10.1007/s00429-009-0221-9 PMC8375673

[19] Gamblin TC, Chen F, Zambrano A, Abraha A, Lagalwar S, et al (2003) Caspase cleavage of tau: linking amyloid and neurofibrillary tangles in Alzheimer's disease. Proc Natl Acad Sci USA 100: 10032–10037. 12888622 10.1073/pnas.1630428100 PMC187753

[20] Rissman RA, Poon WW, Blurton-Jones M, Oddo S, Torp R, et al (2004) Caspase-cleavage of tau is an early event in Alzheimer disease tangle pathology. J Clin Invest 114: 121–130. 15232619 10.1172/JCI20640 PMC437967

[21] Zhang Q, Zhang X, Sun A (2009) Truncated tau at D421 is associated with neurodegeneration and tangle formation in the brain of Alzheimer transgenic models. Acta Neuropathol 117: 687–697. 19190923 10.1007/s00401-009-0491-6

[22] de Calignon A, Fox LM, Pitstick R, Carlson GA, Bacskai BJ, et al (2010) Caspase activation precedes and leads to tangles. Nature 464: 1201–1204. 20357768 10.1038/nature08890 PMC3091360

[23] Vidal R, Barbeito AG, Miravalle L, Ghetti B (2009) Cerebral amyloid angiopathy and parenchymal amyloid deposition in transgenic mice expressing the Danish mutant form of human BRI_2_ . Brain Pathol 19: 58–68. 18410407 10.1111/j.1750-3639.2008.00164.x PMC2605177

[24] Murrell JR, Gnezda AG, Green A, Greally J, Grahame N, et al (2006) Pathology of a Transgenic Mouse Expressing Human P301S Tau. Brain Pathol 16: S44.

[25] Goedert M, Ghetti B, Spillantini MG (2012) Frontotemporal dementia: implications for understanding Alzheimer disease. Cold Spring Harb Perspect Med 2: a006254. 22355793 10.1101/cshperspect.a006254 PMC3281593

[26] Vidal R, Miravalle L, Gao X, Barbeito AG, Baraibar MA, et al (2008) Expression of a mutant form of the ferritin light chain gene induces neurodegeneration and iron overload in transgenic mice. J Neurosci 28: 60–67. 18171923 10.1523/JNEUROSCI.3962-07.2008 PMC2394191

[27] Vidal R, Sammeta N, Garringer HJ, Sambamurti K, Miravalle L, et al (2012) The Psen1-L166P-knock-in mutation leads to amyloid deposition in human wild-type amyloid precursor protein YAC transgenic mice. FASEB J 26: 2899–2910. 22459153 10.1096/fj.12-205542 PMC3382098

[28] Allen B, Ingram E, Takao M, Smith MJ, Jakes R, et al (2002) Abundant tau filaments and nonapoptotic neurodegeneration in transgenic mice expressing human P301S tau protein. J Neurosci 22: 9340–9351. 12417659 10.1523/JNEUROSCI.22-21-09340.2002 PMC6758022

[29] Sahara N, Lewis J, DeTure M, McGowan E, Dickson DW, et al (2002) Assembly of tau in transgenic animals expressing P301L tau: alteration of phosphorylation and solubility. J Neurochem 83: 1498–1508. 12472903 10.1046/j.1471-4159.2002.01241.x

[30] Barbeito AG, Garringer HJ, Baraibar MA, Gao X, Arredondo M, et al (2009) Abnormal iron metabolism and oxidative stress in mice expressing a mutant form of the ferritin light polypeptide gene. J Neurochem 109: 1067–1078. 19519778 10.1111/j.1471-4159.2009.06028.x PMC2696070

[31] Tamayev R, Matsuda S, Fà M, Arancio O, D'Adamio L (2010) Danish dementia mice suggest that loss of function and not the amyloid cascade causes synaptic plasticity and memory deficits. Proc Natl Acad Sci USA 107: 20822–20827. 21098268 10.1073/pnas.1011689107 PMC2996452

[32] Akiyama H, Kondo H, Arai T, Ikeda K, Kato M, et al (2004) Expression of BRI, the normal precursor of the amyloid protein of familial British dementia, in human brain. Acta Neuropathol 107: 53–58. 14586629 10.1007/s00401-003-0783-1

[33] Terry R D, Masliah E, Salmon D P, Butters N, DeTeresa R, et al (1991) Physical basis of cognitive alterations in Alzheimer's disease: synapse loss is the major correlate of cognitive impairment. Ann Neurol 30: 572–580. 1789684 10.1002/ana.410300410

[34] Sze C-I, Troncoso JC, Kawas C, Mouton P, Price DL, et al (1997) Loss of the presynaptic vesicle protein synaptophysin in hippocampus correlates with cognitive decline in Alzheimer disease. J Neuropathol Exp Neurol 56: 933–944. 9258263 10.1097/00005072-199708000-00011

[35] Tsai J, Grutzendler J, Duff K, Gan WB (2004) Fibrillar amyloid deposition leads to local synaptic abnormalities and breakage of neuronal branches. Nat Neurosci 7: 1181–1183. 15475950 10.1038/nn1335

[36] Spires TL, Meyer-Luehmann M, Stern EA, McLean PJ, Skoch J, et al (2005) Dendritic spine abnormalities in amyloid precursor protein transgenic mice demonstrated by gene transfer and intravital multiphoton microscopy. J Neurosci 25: 7278–7287. 16079410 10.1523/JNEUROSCI.1879-05.2005 PMC1820616

[37] Knafo S, Alonso-Nanclares L, Gonzalez-Soriano J, Merino-Serrais P, Fernaud-Espinosa I, et al (2009) Widespread changes in dendritic spines in a model of Alzheimer's disease. Cereb Cortex 19: 586–92. 18632740 10.1093/cercor/bhn111

[38] Dong H, Martin MV, Chambers S, Csernansky JG (2007) Spatial relationship between synapse loss and β-amyloid deposition in Tg2576 mice. J Comp Neurol 500: 311–321. 17111375 10.1002/cne.21176 PMC1661843

[39] Götz J, Chen F, van Dorpe J, Nitsch RM (2001) Formation of neurofibrillary tangles in P301l tau transgenic mice induced by Abeta 42 fibrils. Science 293: 1491–1495. 11520988 10.1126/science.1062097

[40] Lewis J, Dickson DW, Lin WL, Chisholm L, Corral A, et al (2001) Enhanced neurofibrillary degeneration in transgenic mice expressing mutant tau and APP. Science 293: 1487–1491. 11520987 10.1126/science.1058189

[41] Coomaraswamy J, Kilger E, Wölfing H, Schäfer C, Kaeser SA, et al (2010) Modeling familial Danish dementia in mice supports the concept of the amyloid hypothesis of Alzheimer's disease. Proc Natl Acad Sci USA 107: 7969–7974. 20385796 10.1073/pnas.1001056107 PMC2867864

[42] Hurtado DE, Molina-Porcel L, Iba M, Aboagye AK, Paul SM, et al (2010) Aβ accelerates the spatiotemporal progression of tau pathology and augments tau amyloidosis in an Alzheimer mouse model. Am J Pathol 177: 1977–1988. 20802182 10.2353/ajpath.2010.100346 PMC2947292

[43] Zilka N, Korenova M, Novak M (2009) Misfolded tau protein and disease modifying pathways in transgenic rodent models of human tauopathies. Acta Neuropathol 118: 71–86. 19238406 10.1007/s00401-009-0499-y

[44] Delobel P, Lavenir I, Fraser G, Ingram E, Holzer M, et al (2008) Analysis of tau phosphorylation and truncation in a mouse model of human tauopathy. Am J Pathol 172: 123–131. 18079436 10.2353/ajpath.2008.070627 PMC2189621

[45] Spires-Jones TL, de Calignon A, Matsui T, Zehr C, Pitstick R, et al (2008) In vivo imaging reveals dissociation between caspase activation and acute neuronal death in tangle-bearing neurons. J Neurosci 28: 862–867. 18216194 10.1523/JNEUROSCI.3072-08.2008 PMC6670992

[46] Sasaki A, Kawarabayashi T, Murakami T, Matsubara E, Ikeda M, et al (2008) Microglial activation in brain lesions with tau deposits: comparison of human tauopathies and tau transgenic mice TgTauP301L. Brain Res 1214: 159–168. 18457819 10.1016/j.brainres.2008.02.084

[47] Bellucci A, Bugiani O, Ghetti B, Spillantini MG (2011) Presence of reactive microglia and neuroinflammatory mediators in a case of frontotemporal dementia with P301S mutation. Neurodegener Dis 8: 221–829. 21212632 10.1159/000322228 PMC3214942

[48] Yoshiyama Y, Higuchi M, Zhang B, Huang SM, Iwata N, et al (2007) Synapse loss and microglial activation precede tangles in a P301S tauopathy mouse model. Neuron 53: 337–351. 17270732 10.1016/j.neuron.2007.01.010

[49] Ikeda M, Shoji M, Kawarai T, Kawarabayashi T, Matsubara E, et al (2005) Accumulation of filamentous tau in the cerebral cortex of human tau R406W transgenic mice. Am J Pathol 166: 521–531. 15681835 10.1016/S0002-9440(10)62274-2 PMC1602315

[50] Winton MJ, Lee EB, Sun E, Wong MM, Leight S, et al (2011) Intraneuronal APP, not free Aβ peptides in 3xTg-AD mice: implications for tau versus Aβ-mediated Alzheimer neurodegeneration. J Neurosci 31: 7691–7699. 21613482 10.1523/JNEUROSCI.6637-10.2011 PMC3118598

[51] Boutajangout A, Authelet M, Blanchard V, Touchet N, Tremp G, et al (2004) Characterisation of cytoskeletal abnormalities in mice transgenic for wild-type human tau and familial Alzheimer's disease mutants of APP and presenilin-1. Neurobiol Dis 15: 47–60. 14751770 10.1016/j.nbd.2003.09.007

[52] Brendza RP, O'Brien C, Simmons K, McKeel DW, Bales KR, et al (2003) PDAPP; YFP double transgenic mice: a tool to study amyloid-beta associated changes in axonal, dendritic, and synaptic structures. J Comp Neurol 456: 375–383. 12532409 10.1002/cne.10536

[53] Walsh DM, Selkoe DJ (2004) Deciphering the molecular basis of memory failure in Alzheimer's disease. Neuron 44: 181–193. 15450169 10.1016/j.neuron.2004.09.010

[54] Oddo S, Caccamo A, Shepherd JD, Murphy MP, Golde TE, et al (2003) Triple-transgenic model of Alzheimer's disease with plaques and tangles: intracellular Abeta and synaptic dysfunction. Neuron 39: 409–421. 12895417 10.1016/s0896-6273(03)00434-3

[55] Billings LM, Oddo S, Green KN, McGaugh JL, LaFerla FM (2005) Intraneuronal Abeta causes the onset of early Alzheimer's disease-related cognitive deficits in transgenic mice. Neuron 45: 675–688. 15748844 10.1016/j.neuron.2005.01.040

[56] Koffie RM, Meyer-Luehmann M, Hashimoto T, Adams KW, Mielke ML, et al (2009) Oligomeric amyloid beta associates with postsynaptic densities and correlates with excitatory synapse loss near senile plaques. Proc Natl Acad Sci USA 106: 4012–4017. 19228947 10.1073/pnas.0811698106 PMC2656196

[57] Lasagna-Reeves CA, Castillo-Carranza DL, Sengupta U, Clos AL, Jackson GR, Kayed R (2011) Tau oligomers impair memory and induce synaptic and mitochondrial dysfunction in wild-type mice. Mol Neurodegener 6: 39. 21645391 10.1186/1750-1326-6-39 PMC3224595

[58] Matsuda S, Giliberto L, Matsuda Y, Davies P, McGowan E, et al (2005) The familial dementia BRI2 gene binds the Alzheimer gene amyloid-beta precursor protein and inhibits amyloid-beta production. J Biol Chem 280: 28912–28916. 15983050 10.1074/jbc.C500217200

[59] Giliberto L, Matsuda S, Vidal R, D'Adamio L (2009) Generation and initial characterization of FDD knock in mice. PLoS One 4 (11) e7900. 19924302 10.1371/journal.pone.0007900 PMC2774945

[60] Tamayev R, Giliberto L, Li W, d'Abramo C, Arancio O, et al (2010) Memory deficits due to familial British dementia BRI_2_ mutation are caused by loss of BRI_2_ function rather than amyloidosis. J Neurosci 30: 14915–14924. 21048150 10.1523/JNEUROSCI.3917-10.2010 PMC3056548

[61] Tamayev R, Matsuda S, Giliberto L, Arancio O, D'Adamio L (2011) APP heterozygosity averts memory deficit in knockin mice expressing the Danish dementia BRI_2_ mutant. EMBO J 30: 2501–2509. 21587206 10.1038/emboj.2011.161 PMC3116289

