# Supplementary figures and images for: Increased Tau Phosphorylation and Tau Truncation, and Decreased Synaptophysin Levels in Mutant BRI2/Tau Transgenic Mice
Source: PLoS One. 2013 Feb 13;8(2):e56426. doi: 10.1371/journal.pone.0056426 (PMC3572042; doi:10.1371/journal.pone.0056426)

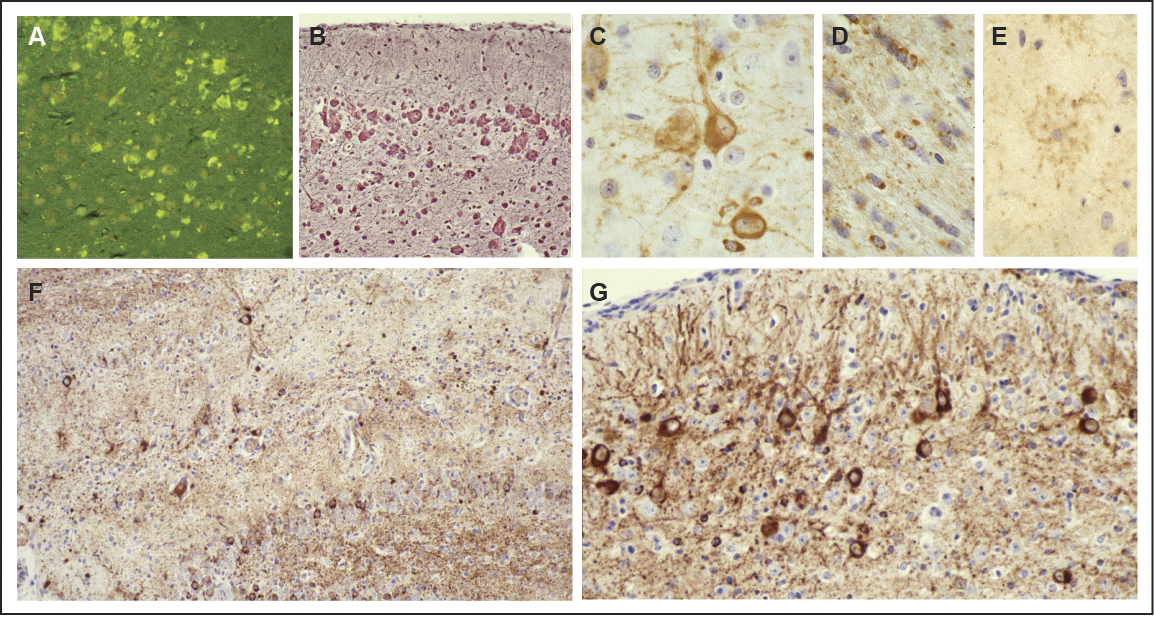

Supplement: Figure S3 — Neuropathologic examination of transgenic mice expressing the P301S mutation (Tg-Tau). ThS-fluorescent (A) and argentophilic (B) deposits in neurons of the frontal and temporal lobes, hippocampus, piriform cortex, brain stem and spinal cord. Neurons of the temporal cortex show argentophilia in the perikaryon extending into cell processes (B). Phosphorylation-dependent anti-tau Abs showed the presence of tau-immunopositive deposits in both nerve (C, neurons of the frontal cortex) and glial (D, oligodendroglial cells of the white matter; E, astrocytes) cells in several areas of the central nervous system with deposition beginning in the temporal lobe, amygdala and hypothalamus. At the age of 5–14 months, tau positive inclusions were observed in the cingulate, somatosensory, motor, and entorhinal cortices as well as the hippocampus, caudate nucleus, putamen, cerebellum, midbrain, pons, medulla, and the anterior and posterior gray horns of the spinal cord (F, hippocampus; G, temporal cortex). Neuronal loss was observed in the temporal lobe, amygdala and hippocampal pyramidal layer. Sections were from a 10 month old homozygous female (A, B), a 12 month old male (C–E), and a 14 month old male (F, G). Immunohistochemistry using Ab AT8 (C–G). Original magnifications 25× (A, F), 40× (B, G), 63× (C–E). (TIF) [file pone.0056426.s003.tif]

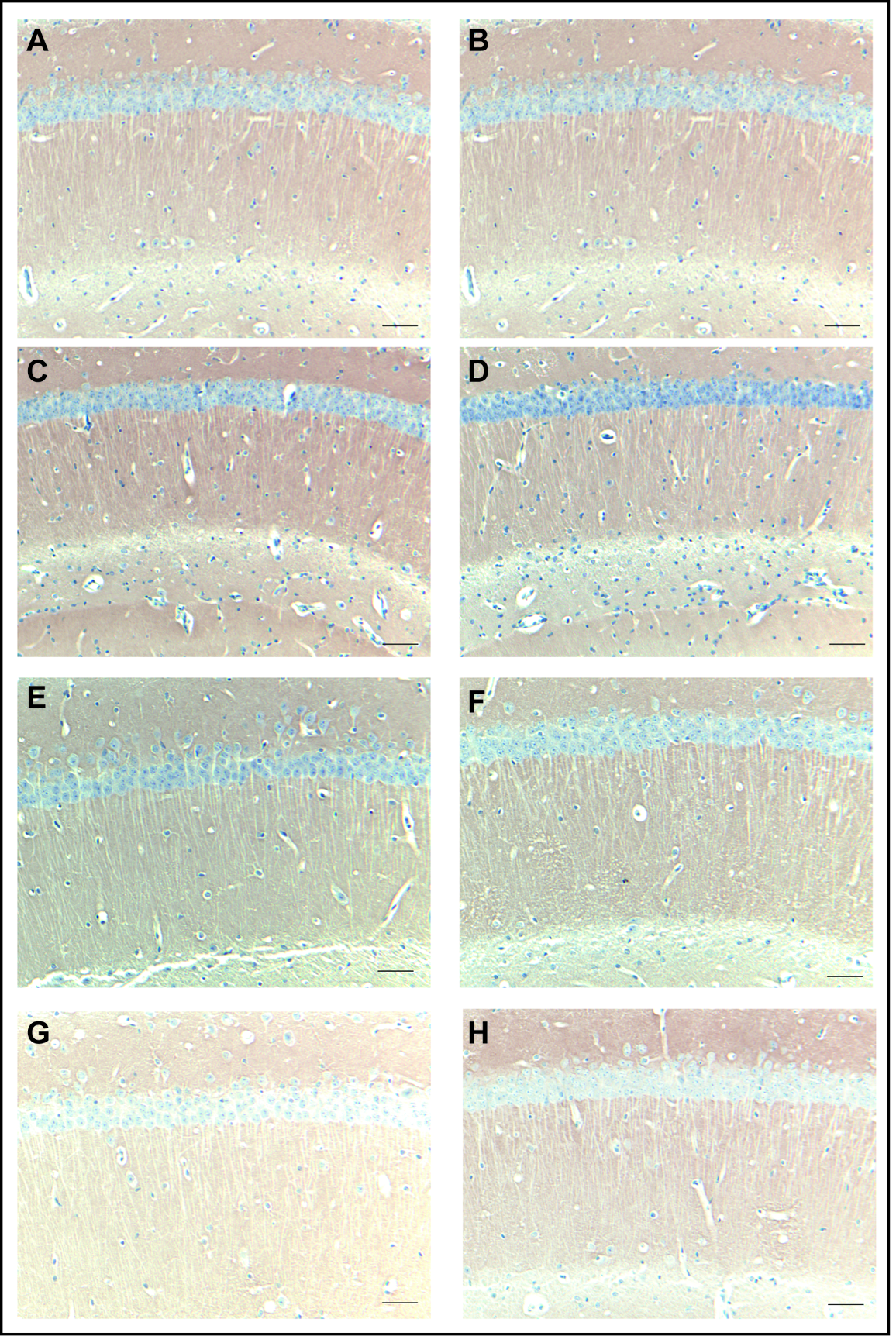

Supplement: Figure S4 — Neuropathologic analysis of synaptophysin in transgenic mice. Representative microphotographs of synaptophysin immunostaining in the hippocampal CA1 region of 6 months old wild-type (A, B), Tg-FDD (C, D), Tg-Tau (E, F), and Tg-FDD-Tau (G, H) mice. Two mice of each genotype are shown. Immunohistochemistry using Ab SY38. Scale bars: A–H, 50 µm. (TIF) [file pone.0056426.s004.tif]
